# Pushing the limits of cardiac MRI: deep-learning based real-time cine imaging in free breathing vs breath hold

**DOI:** 10.1007/s00330-025-11941-2

**Published:** 2025-08-23

**Authors:** Ann-Christin Klemenz, Lena-Maria Watzke, Karolin K. Deyerberg, Benjamin Böttcher, Margarita Gorodezky, Mathias Manzke, Antonia Dalmer, Roberto Lorbeer, Marc-André Weber, Felix G. Meinel

**Affiliations:** 1 Institute of Diagnostic and Interventional Radiology, Pediatric Radiology and Neuroradiology, Rostock University Medical Center, Rostock, Germany; 2 GE HealthCare, Munich, Germany; 3 Department of Radiology, Ludwig-Maximilians University, Munich, Germany

**Keywords:** Cardiac MR, Deep learning, Clinical imaging, Accelerated imaging, Free breathing

## Abstract

**Objective:**

To evaluate deep-learning (DL) based real-time cardiac cine sequences acquired in free breathing (FB) vs breath hold (BH).

**Materials and methods:**

In this prospective single-centre cohort study, 56 healthy adult volunteers were investigated on a 1.5-T MRI scanner. A set of real-time cine sequences, including a short-axis stack, 2-, 3-, and 4-chamber views, was acquired in FB and with BH. A validated DL-based cine sequence acquired over three cardiac cycles served as the reference standard for volumetric results. Subjective image quality (sIQ) was rated by two blinded readers. Volumetric analysis of both ventricles was performed.

**Results:**

sIQ was rated as good to excellent for FB real-time cine images, slightly inferior to BH real-time cine images (*p* < 0.0001). Overall acquisition time for one set of cine sequences was 50% shorter with FB (median 90 vs 180 s, *p* < 0.0001). There were significant differences between the real-time sequences and the reference in left ventricular (LV) end-diastolic volume, LV end-systolic volume, LV stroke volume and LV mass. Nevertheless, BH cine imaging showed excellent correlation with the reference standard, with an intra-class correlation coefficient (ICC) > 0.90 for all parameters except right ventricular ejection fraction (RV EF, ICC = 0.887). With FB cine imaging, correlation with the reference standard was good for LV ejection fraction (LV EF, ICC = 0.825) and RV EF (ICC = 0.824) and excellent (ICC > 0.90) for all other parameters.

**Conclusion:**

DL-based real-time cine imaging is feasible even in FB with good to excellent image quality and acceptable volumetric results in healthy volunteers.

**Key Points:**

***Question*** *Conventional cardiac MR (CMR) cine imaging is challenged by arrhythmias and patients unable to hold their breath, since data is acquired over several heartbeats*.

***Findings*** *DL-based real-time cine imaging is feasible in FB with acceptable volumetric results and reduced acquisition time by 50% compared to real-time breath-hold sequences*.

***Clinical relevance*** *This study fits into the wider goal of increasing the availability of CMR by reducing the complexity, duration of the examination and improving patient comfort and making CMR available even for patients who are unable to hold their breath*.

**Graphical Abstract:**

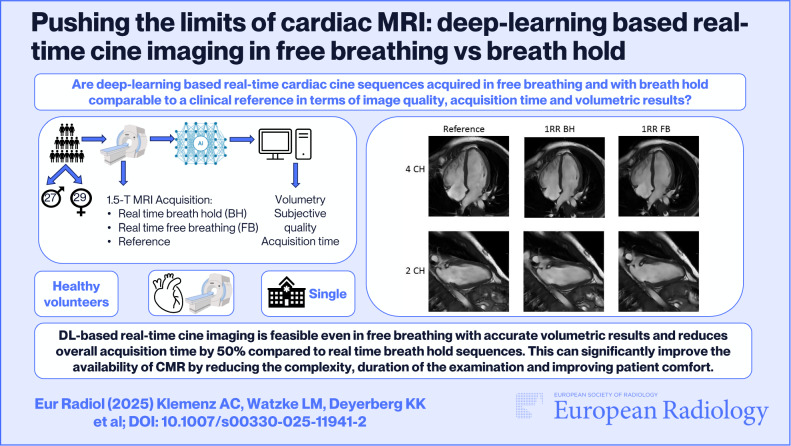

## Introduction

Cardiac magnetic resonance imaging (CMR) is firmly anchored in the clinical routine and is an essential tool for functional and morphological characterisation of the heart. The measurement of ventricular volumes and visual evaluation of cardiac and valvular function by means of cine sequences are well established in clinical routine [1, 2]. Cine sequences create cross-sectional images over multiple heartbeats at different times of the cardiac cycle and can be fitted into a moving image sequence.

Despite extensive technical advancement in the field of cardiovascular MRI, long acquisition times with respiratory manoeuvres and motion artefacts remain major challenges in this imaging modality [3]. Conventional cine imaging is also challenged by arrhythmia, since data is acquired over several heartbeats. In such sequences, k-space data might be acquired at different times of the cardiac cycle, leading to motion artefacts. Image acquisition and reconstruction can be fundamentally improved by neural networks and deep learning (DL)-based algorithms, which are already used for a variety of medical imaging applications [4, 5].

In real-time cine imaging, data for each slice is acquired over a single cardiac cycle. This makes it particularly suitable for patients with arrhythmia as it avoids artefacts created by averaging over multiple cardiac cycles. Until recently, real-time cine imaging was limited by low signal-to-noise ratio and has therefore not seen wide clinical use. Many cardiac imagers only use it as a “last resort” in patients with severe arrhythmia when conventional cine images fail to produce acceptable image quality. However, improved image reconstruction techniques based on DL enable accelerated acquisition with undersampling of the k-space and lead to cine imaging in real time with sufficient image quality [2, 6–8].

With these new DL-based real-time techniques, cine imaging may even be possible without breath holds (BHs). Free-breathing acquisition holds potential to reduce acquisition time, improve patient comfort and make CMR available even for patients who are unable to hold their breath [9, 10]. Therefore, the purpose of our study was to evaluate deep-learning (DL) based real-time cardiac cine sequences acquired in free breathing (FB) vs BH in terms of image quality, acquisition time and volumetric results.

## Material and methods

### Ethical approval and participant selection

This prospective single-centre cohort study was approved by the responsible institutional review board. Written informed consent was received from every subject prior to enrolment.

A total of 56 heart-healthy adult subjects (> 18-years-old) were recruited from among the employees of our University Medical Centre. Male and female volunteers were included in approximately equal numbers to guarantee fair representation of both sexes. Furthermore, age stratification was used to ensure participation of various age groups. Therefore, at least three women and three men were recruited for each of the following age groups: < 34 years, 35–44 years, 45–54 years, > 55 years.

Exclusion criteria were: pregnancy, general contraindications for an MRI examination (e.g. non-MRI-capable implants or claustrophobia), pre-existing conditions that directly or indirectly affect the heart were determined by questionnaire prior to inclusion in the study(e.g. pre-existing diseases of the cardiovascular system, chronic lung diseases, arterial hypertension, diabetes). Demographic data was collected in advance, and the heart rate was recorded by the MRI system.

### MRI protocol

An MRI investigation with a duration of approximately 30 min was carried out on a 1.5-T MRI system (Signa Artist, GE HealthCare) on every participant. Accelerated single-heart-beat (1RR) cine sequences (Sonic DL™, GE HealthCare) with breath-hold commands, followed by the same protocol in FB, were acquired. The protocol included a short-axis stack (SAX) and 2-, 3-, and 4-chamber (2CH, 3CH, and 4CH) views. A DL-based cine sequence acquired with BHs and an acquisition over three cardiac cycles per slice (3RR Sonic DL™) was additionally performed and served as the reference standard for volumetric results. This sequence has been previously successfully validated to produce volumetric results which are not significantly different to conventional (non DL-based) cine sequences [10]. Scan parameter can be found in Supplementary Table S1.

### Subjective image assessment

The subjective image quality of in total of 672 individual cine images (3 different sequences, 4 heart planes, and 56 volunteers) was assessed by two experienced radiologists (both with 5 years of experience in CMR). For this purpose, a software tool developed in-house was used to present images in random order with blinding of acquisition type (FB vs BH). Image quality was rated on a five-point Likert scale (1 = lowest image quality, not diagnostic; 5 = excellent image quality) for each of the following three aspects: absence of artefacts, image sharpness (absence of blurring) and image contrast. The results of the evaluation of all criteria were also combined into an overall subjective quality score. Finally, the subjective image impression of the two readers was averaged.

### Objective image assessment

To objectively quantify image sharpness in cardiac cine MRI data, two established methods are commonly used: the variance of the Laplacian operator and the mean value of the Sobel operator [11–14]. Laplacian-based sharpness assessment utilises the second spatial derivative of the image signal. By convolving the image with the Laplacian kernel

$$\left(\begin{array}{ccc}0 & 1 & 0\\ 1 & -4 & 1\\ 0 & 1 & 0\end{array}\right)$$

regions of rapid intensity change are highlighted. The variance of the Laplacian-filtered image data (σ²) serves as a sharpness metric and is calculated as

$${\sigma }^{2}=\frac{1}{N}{\sum }_{i=1}^{N}(L{(i,j)-\mu })^{2}$$

where *L(i, j)* denotes the Laplacian-filtered pixel values and *μ* their mean. Higher variance values correlate with better edge-selective image quality.

In parallel, the Sobel operator is used as a gradient-based measure. Using 3 × 3 convolution matrices for horizontal (*Gx*) and vertical (*G*_*γ*_) gradients, image sharpness is quantified via the mean value of the resulting gradient magnitude:

$$|\bar{G}|=\frac{1}{N}\sum \sqrt{G{{{{\rm{x}}}}}^{2}+{{G}_{\gamma }}^{2}}$$

This method is sensitive to edge strength and noise, with higher mean values indicating sharper structural contours. In combined approaches, Laplacian variance enables detection of local sharpness variations, while the Sobel mean captures global contour information. Both sharpness assessments were done for all acquired cine images and cine image stacks of the 2CH, 3CH, and 4CH views and the short axis.

### Assessment of acquisition time

For a subset of 37 volunteers, the duration of the FB and BH protocol was manually stopped with a stopwatch from the beginning of the acquisition until the end of the protocol, containing a SAX, 2CH, 3CH, and 4CH cine sequence. For BH sequences, this included additionally the total runtime of the sequences, BHs, and the pauses between the BHs.

### Post-processing and volumetric results

For volumetric assessment, a standard commercial software (cvi42 version 5.16, Circle Cardiovascular Imaging Inc., Calgary, Canada) was utilised. The software automatically delineated the endocardial and epicardial contours. Papillary muscles were not segmented and incorporated into the blood pool. Subsequently, all contours were reviewed and revised by an experienced cardiovascular radiologist (5 years of experience). Volumetric parameters of the left (LV) and the right ventricle (RV) (end-diastolic volume (EDV), end-systolic volume (ESV), stroke volume (SV), ejection fraction (EF) and LV mass) were obtained.

### Statistical analysis

Demographic characteristics and ventricular volumetric results of the study sample are summarised by median and range (minimum to maximum) since the Shapiro-Wilk test for normal distribution revealed, that most of the parameters did not pass normality testing. Subjective image quality is presented as median per reader and as overall median score over both readers and all quality parameters. Objective and subjective quality differences between BH and FB sequences were investigated by the Wilcoxon matched-pairs signed rank sum test. In addition, quality differences were displayed in bar charts. Differences between reference, 1RR BH and 1RR FB in volumetric results were assessed by the Wilcoxon matched-pairs signed rank sum test. Bland–Altman analysis was used to demonstrate absolute mean differences of volumetric results together with 95% limits of agreement. Scatter plots, together with identity lines, were used to show correlations between parameters. For assessing the accordance of all cine sequences, the intra-class-correlation coefficients (ICC) from two-way random-effects models were calculated from the volumetric results of the SAX stack. *p*-values < 0.05 were considered statistically significant. Statistical analyses were performed using Stata 18.0 (Stata Corporation) and GraphPad Prism (version 10.3.0; GraphPad Software, LLC).

## Results

### Demographic characteristics

In total, 56 healthy volunteers (male: *n* = 27; female: *n* = 29) participated in the study. Median age of all volunteers was 52 years (range: 23–68 years), median weight was 77 kg (range: 50–100 kg), and median height was 1.75 m (range: 1.59–1.96 m). The heart rate was recorded by the MRI system and corresponded to 68 bpm (range: 44–101 bpm). Detailed characteristics stratified by sex can be found in Table 1.

**Table 1:** Participant characteristics shown as median and interquartile range

	All participants (*n* = 56)	Men (*n* = 27)	Women (*n* = 29)
Demographic parameters			
Age [years]	42 (27–55)	44 (32–56)	40 (25–53)
Weight [kg]	76 (67–87)	85 (77–92)	67 (60–76)
Height [m]	1.75 (1.69–1.81)	1.82 (1.76–1.90)	1.69 (1.63–1.72)
BMI [kg/m^2^]	24.8 (22.6–27.1)	25.1 (23.7–27.1)	23.5 (22.2–26)
Heart rate [min^−^^1^]	68 (60–78)	66 (58–74)	68 (65–80)

### Subjective image quality

**Fig. 1:**
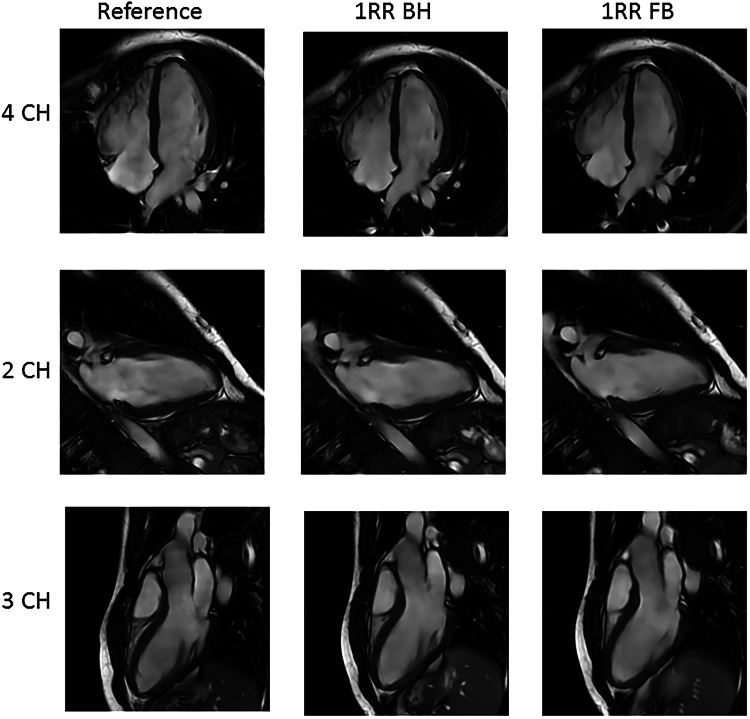
Image examples of a 23-year-old male from 2CH, 3CH and 4CH views acquired with accelerated sequences (1RR) in FB and with BH and reference sequence

**Fig. 2:**
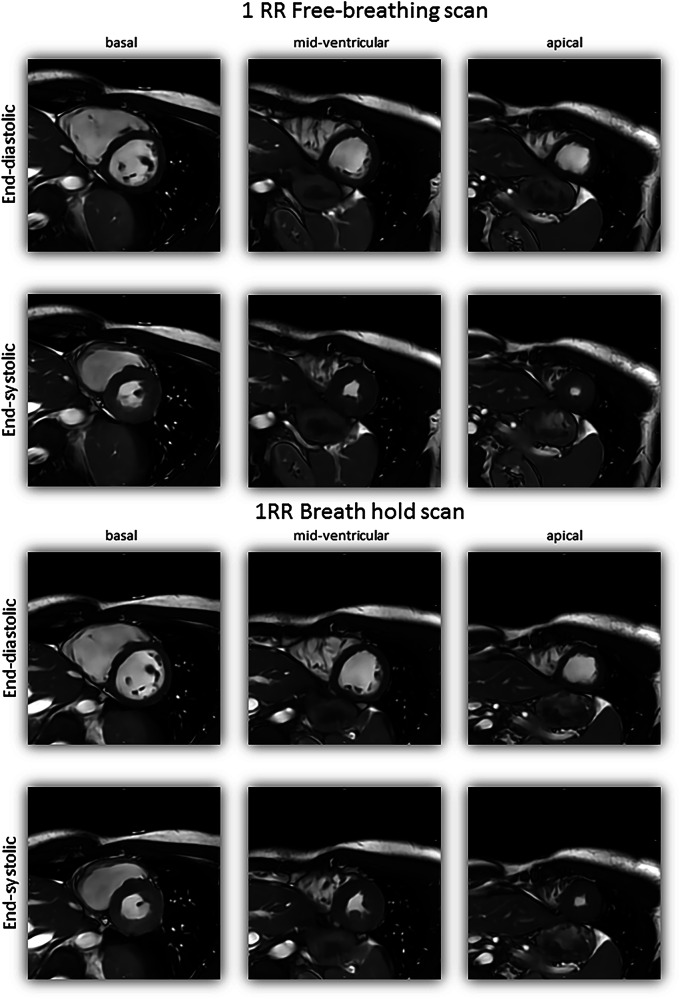
Examples for the subjective image quality of basal, mid-ventricular and apical slices of the SAX stack in the end-diastolic and end-systolic cardiac phases from the same 23-year-old male volunteer

Image examples for the reference sequence, 1RR BH and 1RR FB, can be found in Fig. 1 for the long-axis views and image examples for the SAX stack 1RR BH and 1RR FB in Fig. 2. Fine structures such as trabecula and atrioventricular valves are preserved in the accelerated 1RR BH and 1RR FB sequences. Video files are available as supplementary material. Examples for different subjective image impressions and the corresponding rating can be found in Fig. S1.

**Fig. 3:**
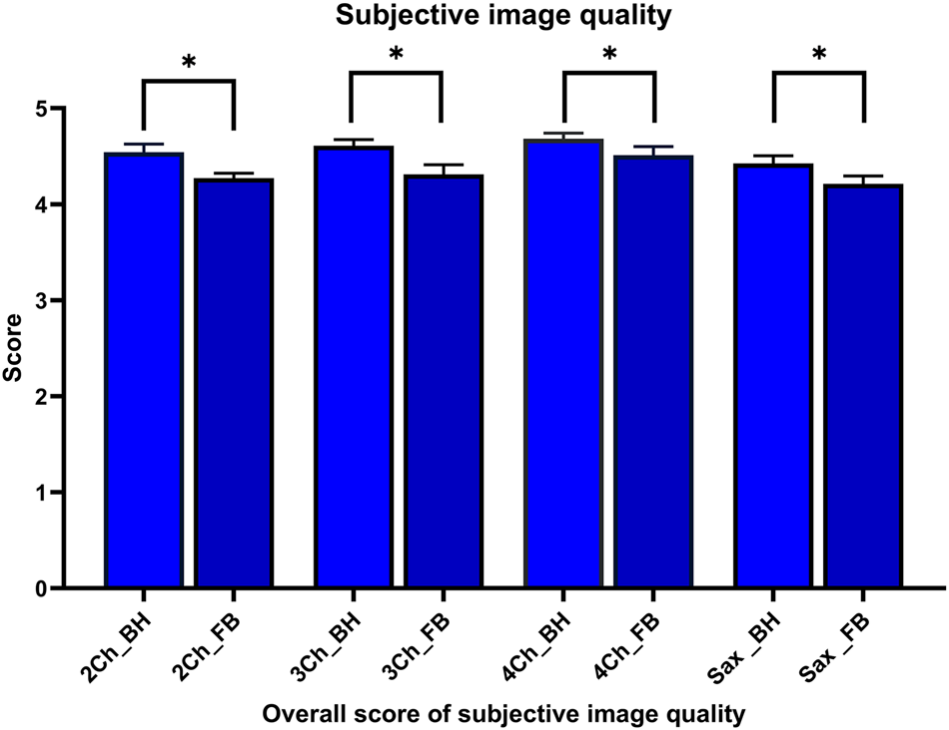
Overall score of subjective image quality from 2CH, 3CH, 4CH and short axis in acquired FB and with BH. *p < 0.001 from Wilcoxon paired signed rank sum test

A mean overall score for the 2CH, 3CH, 4CH view and the SAX stack of the BH cines of 4.5 (range: 3–5), 4.6 (range: 3.3–5), 4.7 (range: 3–5), and 4.4 (range: 3–5), respectively, was detected. The same cine sequences in FB had a mean overall score of 4.3 (range: 3–5), 4.3 (range: 2.7–5), 4.5 (range: 2.7–5), and 4.2 (range: 2.7–5), respectively (Fig. 3), which was significantly different to all overall scores in BH. The main differences, as well as the lowest scores, between BH and FB cines occurred in the category “image sharpness (absence of blurring)” for all heart planes. Detailed ratings of the subjective image quality are given in Table 2.

**Table 2:** Subjective image quality of all heart planes in the categories contrast, blurring and artefacts

	Subjective image quality of BH sequences			Subjective image quality of FB sequences			
	Reader 1 (Min; Max)	Reader 2 (Min; Max)	Overall score (Min; Max)	Reader 1 (Min; Max)	Reader 2 (Min; Max)	Overall score (Min; Max)	*p*-Value
2CH 1RR			4.6 (3; 5)			4.3 (3; 5)	< 0.001
Contrast	5 (3; 5)	5 (4; 5)	5 (3; 5)	5 (3; 5)	4.5 (3; 5)	5 (3; 5)	0.038
Sharpness	4 (2; 5)	4 (3; 5)	4 (2; 5)	4 (1; 5)	4 (3; 5)	4 (1; 5)	< 0.001
Absence of artefacts	5 (2; 5)	5 (4; 5)	5 (2; 5)	5 (2; 5)	4 (3; 5)	4 (2; 5)	< 0.001
3CH 1RR			4.6 (3; 5)			4.3 (2; 5)	< 0.001
Contrast	5 (4; 5)	5 (4; 5)	5 (4; 5)	5 (3; 5)	5 (4; 5)	5 (3; 5)	0.002
Sharpness	4 (2; 5)	4 (4; 5)	4 (2; 5)	4 (1; 5)	4 (3; 5)	4 (1; 5)	< 0.001
Absence of artefacts	5 (4; 5)	5 (4; 5)	5 (4; 5)	5 (2; 5)	4.5 (3; 5)	5 (2; 5)	< 0.001
4CH 1RR			4.6 (3; 5)			4.6 (2; 5)	< 0.001
Contrast	5 (5; 4)	5 (4; 5)	5 (4; 5)	5 (5; 4)	5 (4; 5)	5 (4; 5)	0.006
Sharpness	4 (2; 5)	4 (4; 5)	4 (2; 5)	4 (1; 5)	4 (3; 5)	4 (1; 5)	0.006
Absence of artefacts	5 (2; 5)	5 (4; 5)	5 (2; 5)	5 (3; 5)	5 (3; 5)	5 (3; 5)	0.014
Sax 1RR			4.6 (3; 5)			4.3 (2; 5)	< 0.001
Contrast	5 (4; 5)	5 (4; 5)	5 (4; 5)	5 (4; 5)	5 (4; 5)	5 (4; 5)	0.359
Sharpness	4 (2; 5)	4 (3; 5)	4 (2; 5)	3 (2; 5)	4 (3; 5)	4 (2; 5)	< 0.001
Absence of artefacts	4 (2; 5)	5 (3; 5)	4 (2; 5)	4 (2; 5)	4 (3; 5)	4 (2; 5)	< 0.001

Displayed are the individual values of 2CH, 3CH, 4CH views and SAX for reader 1 and reader 2 of BH and FB sequences as median values + range (min to max). *p*-values are from the Wilcoxon paired signed rank sum test evaluating differences between the overall scores of BH and FB sequences

### Objective image quality

**Fig. 4:**
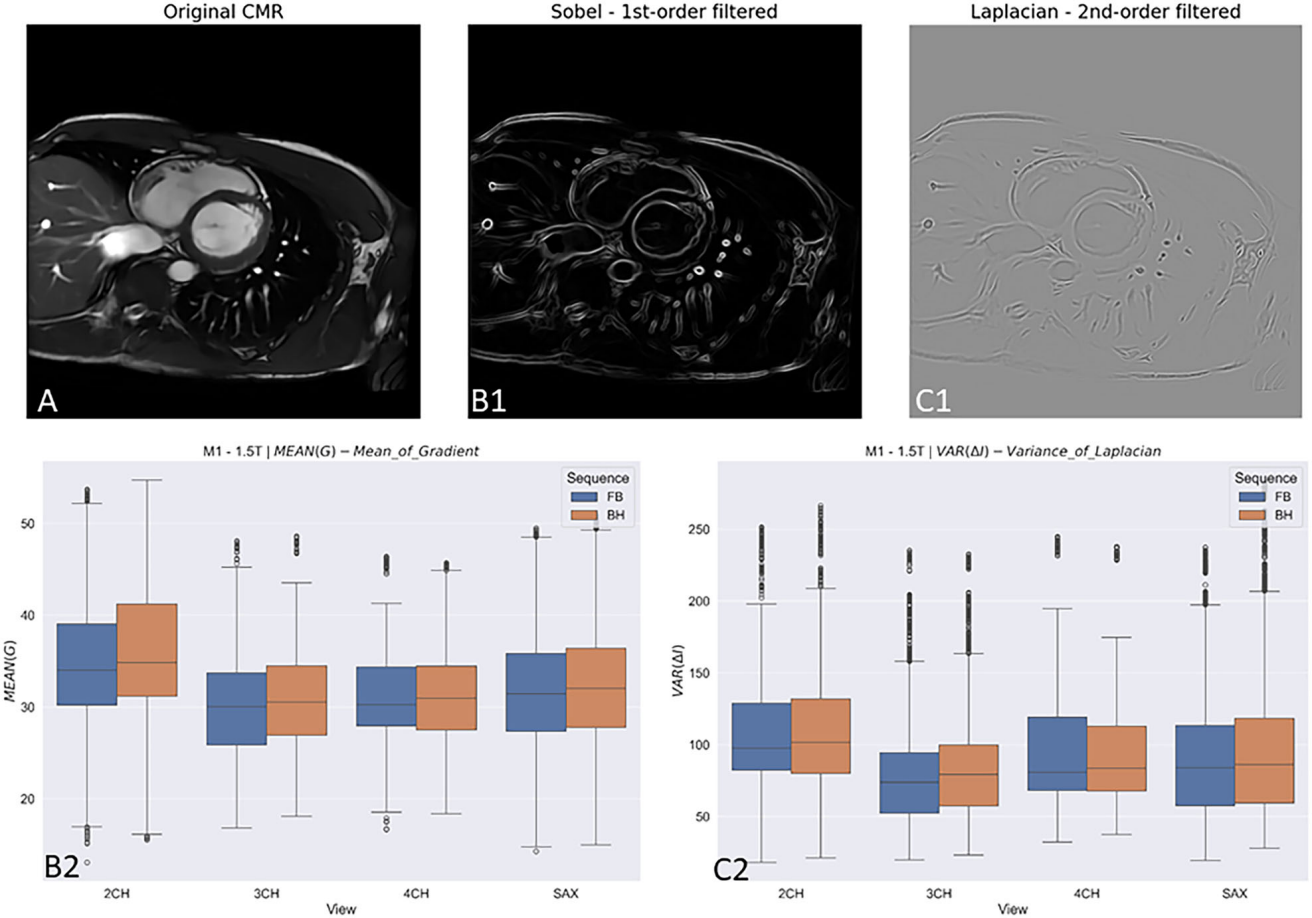
Objective image quality assessment with the Mean of 1st-order filtered and 2nd-order filtered images. The upper part of the image shows an original SAX image (**A**) and an example of the first-order filtered Sobel operator (**B1**) and the second-order filtered Laplacian variance (**C1**). The corresponding boxplots of all acquired images analysed by heart plane (2CH, 3CH, 4CH view, and SAX) and BH and FB sequence are displayed in **B2** (mean of the Sobel operator) and **C2** (variance of Laplacian). Box plots show the minimum to maximum distribution. Outliers are displayed as dots

The Laplacian-based sharpness assessment revealed statistically significant differences of the 2CH view (median: 101.7 (BH) vs 97.7 (FB)), 3CH view (median: 79.3 (BH) vs 73.9 (FB)) and SAX (median: 86.3 (BH) vs 84.0 (FB)). Only the 4CH view did not show significant differences in the Laplacian-based sharpness parameter, with a *p* = 0.76 and a median of 83.7 (BH) and 80.9 (FB). The analysis of the Sobel operator showed the same results with significant differences of the 2CH view, 3CH view and SAX and no significant difference of the 4CH view. The results are plotted in Fig. 4.

### Objective comparison of the BH and FB protocols

#### Acquisition time

Overall acquisition time (including sequence run time, time needed for breathing commands and “recovery” breaths between breathing commands) for a complete set of cine images (2CH, 3CH, 4CH view, and SAX stack) was significantly lower with the FB protocol (median: 90 s, range: 64–140 s) compared to the BH protocol (median: 180 s, range: 123–270 s, *p* < 0.001).

#### Volumetric results

Volumetric analysis was done on the basis of the SAX acquired by the clinical reference sequence, the 1RR BH sequence and the 1RR FB sequence. All absolute volumetric results of the left and the RV can be found in Table 3. In median, left ventricular EDV and ESV were minimally underestimated by the 1RR BH sequence (by 1 mL) and slightly overestimated by the 1RR FB sequence (by 3 mL) compared to the reference standard. A similar trend was detected in the right ventricular analysis.

**Table 3:** Volumetric results of the reference, 1RR BH, and 1RR FB sequence shown as median and range

Volumetric results	Reference			1RR Cine BH			1RR Cine FB		
Left ventricle	Median	Range (min/max)		Median	Range (min/max)		Median	Range (min/max)	
LV EDV (mL)	153	102	245	152	100	231	155	98	233
LV ESV (mL)	64	36	103	63	36	104	66	45	109
LV SV (mL)	89	62	150	88	61	148	85	53	152
LV EF (%)	59	50	69	60	49	69	59	46	69
LV mass (g)	97	59	162	94	58	159	96	57	162
RV									
RV EDV (mL)	171	97	289	169	98	284	172	94	284
RV ESV (mL)	74	37	142	79	39	145	79	40	137
RV SV (mL)	88	57	148	89	58	141	86	54	147
RV EF (%)	55	42	66	55	45	64	53	40	65

*LV* left ventricular, *RV* right ventricular, *EDV* end-diastolic volume, *ESV* end-systolic volume, *SV* stroke volume, *EF* ejection fraction

The statistical analysis of all sequences showed significant differences between the accelerated 1RR BH sequences and the reference in LV EDV, LV ESV, LV SV and LV mass with a median of differences of 2.3 mL, 1.2 mL, 1.8 mL and 1.3 g, respectively. The comparison of the reference and the 1RR FB sequence revealed a significant median difference of LV EDV, LV SV and LV EF with 3.6 mL, 3.4 mL and 1.6%, respectively. 1RR in BH and in FB only differed significantly in LV EF (median of difference 1.3%). Overall, the 1RR FB sequence showed slightly higher deviations (medians of differences) from the reference sequence than the 1RR BH sequence. All medians of differences and the corresponding *p*-values are displayed in Table 4. The volumetric results, including the significances, are also displayed as boxplots in Fig. S2 for the left ventricle (LV) and in Fig. S3 for the RV.

**Table 4:** Comparison of volumetric results of reference, 1RR BH and 1RR FB sequences

Volumetric results	Reference vs 1RR BH		Reference vs 1RR FB		1RR BH vs 1RR FB	
LV	Median of differences (IQR)	*p*-value	Median of differences (IQR)	*p*-value	Median of differences (IQR)	*p*-value
LV EDV (mL)	2.3 (−0.1; 5.4)	< 0.001	3.6 (−2.4; 7.0)	0.040	−0.4 (−5.9; 6.2)	0.760
LV ESV (mL)	1.2 (−0.9; 2.7)	0.022	−0.8 (−4.8; 3.8)	0.338	−0.6 (−7.0; 2.5)	0.069
LV SV (mL)	1.8 (−1.4; 3.6)	0.010	3.4 (−1.7; 7.6)	0.002	0.6 (−2.8; 4.3)	0.137
LV EF (%)	−0.1 (−1.0; 0.9)	0.903	1.6 (−1.3; 3.4)	0.018	1.3 (−0.7; 3.7)	0.006
LV mass (g)	1.3 (−0.8; 3.1)	0.006	1.1 (−1.1; 3.1)	0.064	−0.4 (−3.3; 2.3)	0.381

RV	Median of differences (IQR)	*p*-value	Median of differences (IQR)	*p*-value	Median of differences (IQR)	*p*-value
RV EDV (mL)	1.5 (−3.5; 5.0)	0.326	−1.3 (−10.2; 4.5)	0.173	−2.6 (−10.6; 3.9)	0.108
RV ESV (mL)	−0.8 (−4.8; 2.5)	0.188	−3.7 (−10.1; 1.1)	< 0.001	−2.4 (−8.9; 1.4)	0.005
RV SV (mL)	2.5 (−1.8; 6.3)	0.029	3.6 (−3.4; 7.5)	0.020	0.2 (−5.3; 5.2)	0.625
RV EF (%)	1.0 (−1.0; 2.7)	0.016	1.7 (−0.2; 4.8)	< 0.001	1.3 (−1.2; 3.0)	0.013

Shown are the medians of differences, the interquartile range, and the *p*-values by the Wilcoxon paired signed rank sum test

*LV* left ventricular, *RV* right ventricular, *EDV* end-diastolic volume, *ESV* end-systolic volume, *SV* stroke volume, *EF* ejection fraction

The Bland–Altman analysis revealed the lowest bias for all volumetric parameters for the comparison of reference and 1RR BH sequence. The 95% limits of agreement were wider for reference vs 1RR FB (LV EDV: −15.6 to 19.9; LV ESV: −15.3 to 13.1) compared to reference vs 1RR BH (LV EDV: −8.6 to 14.75; LV ESV: −7.1 to 9.6). The limits of agreement were wider for all RV volumetric parameters compared to LV parameters. Details of the Bland–Altman analysis can be found in Table S1. For LV EDV; LV ESV; LV SV, and LV EF, the results were also visualised as plots in the Supplemental Material (Fig. S4).

#### Correlation analysis

**Fig. 5:**
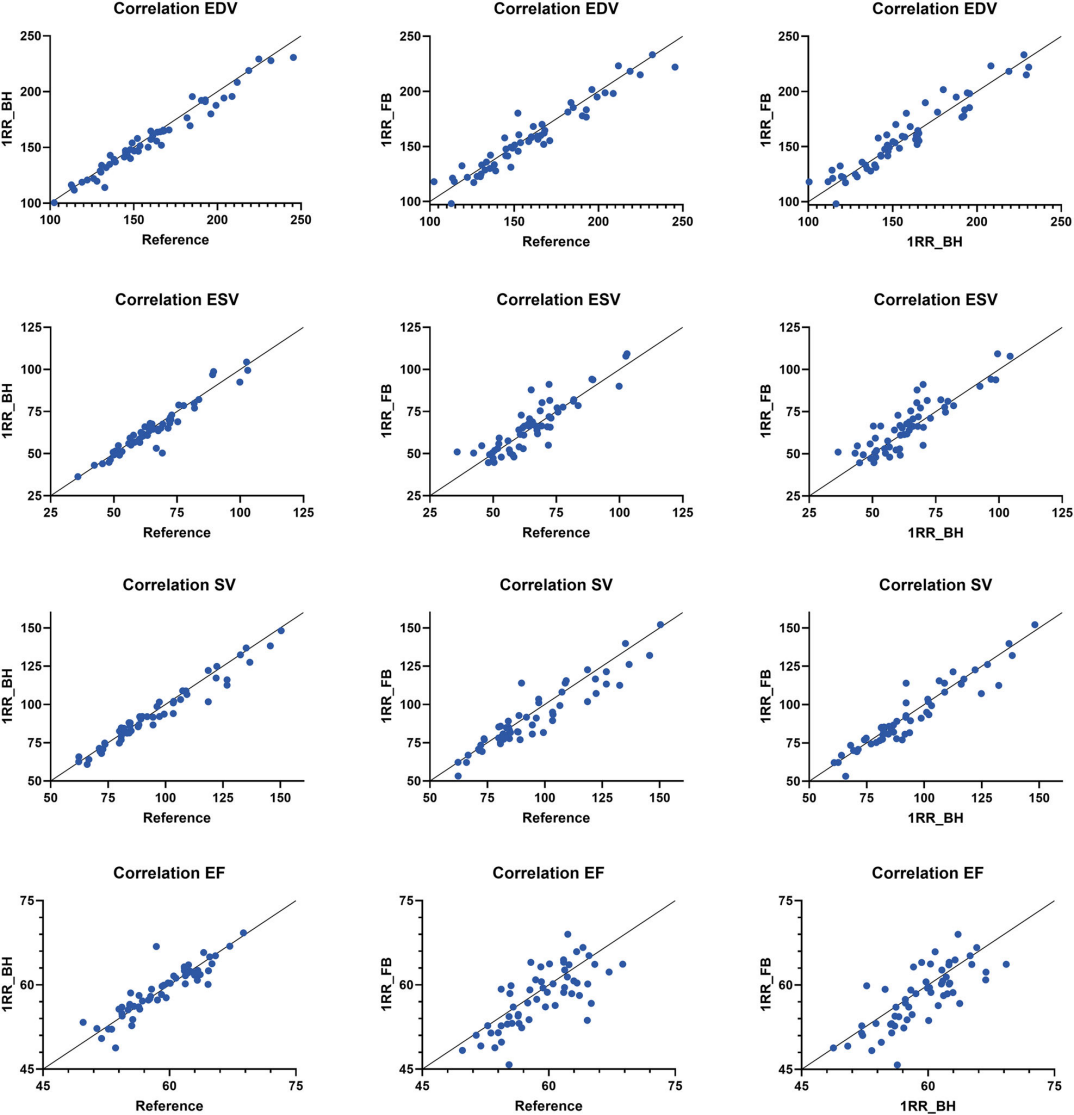
Correlation analysis of 1RR_BH vs reference, 1RR_FB vs reference and 1RR_BH vs 1RR_FB of left ventricular volumetric parameter. EDV, end-diastolic volume; ESV, end-systolic volume; SV, stroke volume; EF, ejection fraction

Correlation plots of the volumetric results (LV EDV, LV ESV; LV SV and LV EF) of the reference, 1 RR BH and 1RR FB are shown in Fig. 5. A line of identity is plotted for better visualisation of related values. Very strong correlations can be found between reference and 1RR BH results, with only minimal differences in all volumetric parameters. The reference vs 1RR FB results showed an increased scattering and small deviations, with a trend of underestimating the volumetric results in the 1RR FB compared to the reference. This effect was stronger for SV and EF due to the combined effect in EDV and ESV. The correlation of 1RR BH and 1RR FB shows underestimation of volumetric results in the 1RR FB sequence. These results correspond with the Bland–Altman analysis.

For a more detailed correlation analysis, the ICC values for all LV and RV parameters can be found in Table 5. The ICC analysis demonstrated excellent agreement of reference and 1RR BH values (ICC > 0.90), with the only exception of RV EF (good agreement, ICC = 0.887). For 1RR FB, ICCs indicated good agreement with reference and 1RR BH sequences for LV and RV EF (ICC 0.825/0.824 and 0.847/0.869, respectively) and excellent correlation for all other parameters.

**Table 5:** Intraclass correlation coefficient for all left ventricular (LV) and right ventricular (RV) parameters of reference, 1RR BH and 1RR FB SAX

ICC	Reference vs 1RR BH	Reference vs 1RR FB	1RR BH vs 1RR FB
LV			
LV EDV (mL)	0.989	0.978	0.975
LV ESV (mL)	0.976	0.938	0.929
LV SV (mL)	0.987	0.960	0.971
LV EF (%)	0.950	0.825	0.847
RV			
RV EDV (mL)	0.986	0.983	0.979
RV ESV (mL)	0.972	0.960	0.954
RV SV (mL)	0.973	0.955	0.965
RV EF (%)	0.887	0.824	0.869

*EDV* end-diastolic volume, *ESV* end-systolic volume, *SV* stroke volume, *EF* ejection fraction, *BH* breath hold, *FB* free breathing

Intraclass correlation coefficients (ICC) are from a two-way random-effects model

#### Exemplary case report

**Fig. 6:**
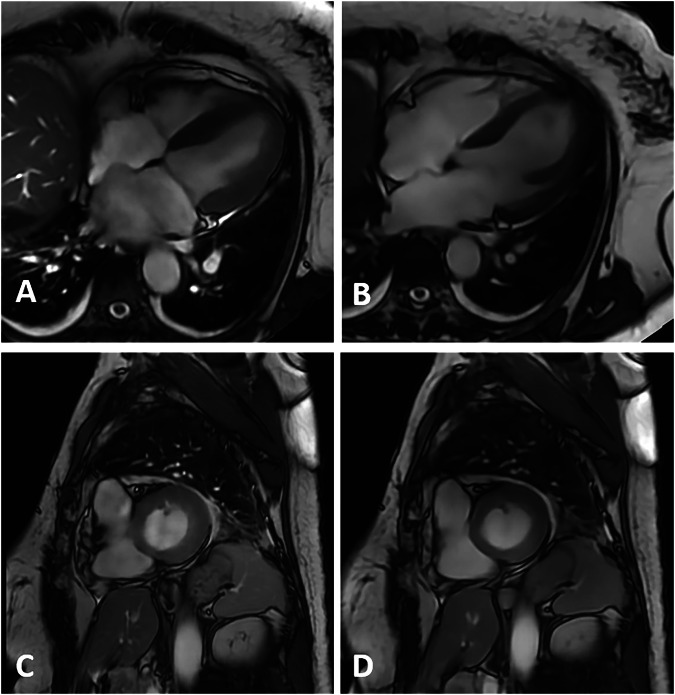
Image examples of a 52-year-old female patient with sudden cardiac arrest. Displayed are a SAX image acquired at the end-systolic phase (**C**, **D**) and 4CH views (**A**, **B**). **A**, **C** Accelerated DL cine sequence acquired over three heartbeats with breathholding. The displayed SAX stack was aborted in midventricular slice position due to frequent arrhythmia. The image quality was compromised by the presence of arrhythmia, which resulted in an increase in blurring. **B**, **D** Accelerated DL cine sequence acquired over a single heartbeat under FB. The complete SAX stack was completed in the first attempt with sufficient image quality. The 4CH view achieved a sufficient diagnostic image quality for visualisation of the cardiac structures, including an atrial septum aneurysm

A 52-year-old female patient with a history of breast cancer was transferred to the emergency department (ER) of our university medical centre after a sudden cardiac arrest. Cardiopulmonary resuscitation was performed by emergency medical services, and a return of spontaneous circulation was achieved. Initial computed tomography ruled out pulmonary embolism. Bedside transthoracal echocardiography showed only a mildly reduced EF and no major wall motion abnormalities. Invasive coronary angiography was performed due to raised levels of troponin, and obstructive coronary artery disease was ruled out. Five days after the initial cardiac arrest, a cardiac MRI scan was performed for further cardiac evaluation, particularly to rule out cardiac fibrosis after radiation therapy or other structural heart diseases. On the day of examination, the patient showed short episodes of bigeminy and persistent premature ventricular complexes in the electrocardiogram. Clinical standard MRI cine sequences for long and short axis views could not be successfully completed due to arrhythmia. Therefore, the MRI scan was performed utilising novel DL cine sequences, including single heartbeat acquisition in FB. Image examples of this patient are shown in Fig. 6.

## Discussion

Only a few prospective studies examining accelerated DL-based sequences in BH and FB have been conducted so far. This prospective cohort study in healthy volunteers compared DL-based real-time cine sequences (BH and FB) with a validated reference sequence regarding image quality, acquisition time, as well as left and right ventricular volumetric results. We demonstrated that DL-based real-time cine imaging is feasible even in FB with good to excellent image quality. Compared to the real-time cine sequence acquired with BHs, performing real-time cine imaging in free-breathing acquisition further reduces overall acquisition time by 50%. The correlation of volumetric results with the reference standard is slightly weaker for free-breathing real-time cine compared to breath-hold real-time cine images, but accuracy is still high.

### Time-saving potential

A central and novel finding of our study is that there is substantial additional potential for time saving if real-time cine sequences are acquired in FB. Even though the signal acquisition time is equal, free-breathing acquisition allows for all cine sequences to be performed in immediate succession and saves the time required for breathing commands, as well as “recovery” breaths between BHs. In our cohort, this cut median total acquisition time for long-axis and SAX cine sequences by half from 180 s to 90 s. This time-saving potential comes on top of the substantial time saving that comes with DL-based real-time sequences compared to non-DL-based clinical standard sequences, which are typically acquired over 8–12 cardiac cycles per slice [10].

#### Image quality

Earlier studies using free-breathing real-time cine sequences not based on DL networks resulted in good volumetric correlation to reference sequences but reduced image quality [15, 16]. The DL-based real-time cine sequence evaluated in our study uses DL based image reconstruction to calculate high-quality images from severely undersampled raw data. Previous studies have shown that the image quality of DL-based real-time cine sequences remained very good (albeit slightly inferior to standard sequences with much longer acquisition times [10]). In the present study, we observed that the image quality of DL-based real-time cine sequences was superior with BHs. There was a reduction in image quality with FB acquisition (primarily driven by artefacts and reduced image sharpness), but image quality remained good to excellent even with FB real-time acquisition.

In our study, we rated image quality by three pre-specified criteria: image contrast, image sharpness (absence of blurring), and absence of artefacts. In the real clinical world, the required image quality for cine images may also depend on the specific diagnostic task and clinical question. For example, blurring with reduced image sharpness may not be a problem for volumetric analysis and assessment of regional wall motion abnormalities but may preclude detection of subtle valvular regurgitation or a small valvular mass. Thus, while DL-based real-time cine sequences in FB may provide sufficient image quality for the majority of patients and indications for cardiac MRI, we recommend that cardiac imaging professionals adjust the balance between time efficiency and image quality to their specific patients and needs.

#### Volumetric differences

DL-based accelerated, undersampled cine sequences from the same vendor were already used in previous studies [7, 8, 10, 17]. They revealed no major impact of DL based sequences compared to non-DL sequences, but in two studies, the highly accelerated real-time cine with BHs showed significant volumetric differences to the 3RR cine sequence [8, 10].

Revealing the potential of DL image reconstruction, Zucker et al reported first results of the same accelerated DL cine sequences in a free-breathing setting prior to clinical use [18]. The ICC of LV EDV and LV ESV between the reference sequence and FB was comparable to our results, whereas our results for the ICC for LV EF are higher.

Even though the volumetric agreement within all sequences was high, some deviations and outliers were detected. Those volumetric differences between the sequences might have been caused by different reasons. Due to the blinded post-processing analysis, SAX slices that were close to the valve level can be included or excluded from the volumetric analysis. In borderline cases, the blinded analysis might therefore lead to different volumetric results due to the inclusion or exclusion of the basal slices. Furthermore, in a few cases, the volunteers have slightly moved during the exam, and therefore, small inconsistencies in slice positions have occurred within the same exam, leading to a deviation of volumes. Another reason was a respiratory mis-triggering due to the irregular breathing of the volunteers. Lastly, smaller differences occurred as a result of the blurring of the 1RR sequences, leading to different contouring.

From a clinical perspective, differences of < 10% in EF and < 20 mL in volumes of the LV are generally considered acceptable, and differences of this magnitude would rarely change clinical decision-making. The 95% limits of agreement reported in our cohort are within this range. The volumetric analysis of the RV is somewhat less reproducible compared to the LV. We did observe some cases with differences exceeding 20 mL for RV volumes in the free-breathing sequence. Thus, we would not recommend this particular sequence in cases where clinical decision making is based on precise quantification of RV function.

Our results are in line with a prospective study comparing accelerated DL-based FB to standard BH cine sequences in patients with ischaemic heart disease, showing only a small bias in LV EF, LV ESV and LV EDV between the sequences [19]. Our study in healthy volunteers goes beyond these previous investigations, as we also performed RV analysis and directly compared real-time cine sequence with and without BH.

#### Clinical relevance and outlook

This study fits into the wider goal of increasing the availability of cardiac MRI by reducing the complexity and duration of the examination and improving patient comfort. In combination with free-breathing single-shot late enhancement sequences [20–22], the free-breathing real-time cine sequences used in our study may allow for a comprehensive CMR examination to be performed completely in FB and in less than 10 min. This may be especially useful in claustrophobic patients or in patients who are unable to hold their breath. With this new clinical reality, the rate-determining factor for the cardiac MRI workflow will no longer be sequence run time but rather patient preparation and sequence planning by the operating technician. Thus, emerging applications of artificial intelligence in cardiovascular MRI are likely to focus on streamlining the workflow both outside and inside the scanner room. For example, AI-based fully automated plane prescription may reduce the time required for positioning of cardiac imaging planes.

#### Limitations

We evaluated accelerated sequences from one vendor at one magnet field strength in a single-centre study. Furthermore, we examined healthy volunteers and not patients with different cardiac conditions. We would expect that the difference in image quality between breath-hold and FB scans may be even smaller in patients, who may not be able to hold their breath, as well as healthy participants.

## Conclusion

DL-based real-time cine imaging is feasible even in FB, with slightly reduced but still good to excellent image quality in heart-healthy volunteers. Volumetric results showed only a few significant but clinically irrelevant differences between the reference and real-time acquisition with and without breath manoeuvre. Compared to the real-time cine sequence acquired with BHs, performing real-time cine imaging in free-breathing acquisition further reduces overall acquisition time by 50%. This represents a major step towards an ultra-short CMR protocol acquired completely in FB.

## Supplementary information

ELECTRONIC SUPPLEMENTARY MATERIAL

Video 1

Video 2

Video 3

Video 4

Video 5

Video 6

Video 7

Video 8

## Data Availability

All data generated or analysed during the study are available from the corresponding author by request.

## Abbreviations

2CH: 2-Chamber-view
3CH: 3-Chamber-view
4CH: 4-Chamber-view
BH: Breath hold
CMR: Cardiac magnetic resonance imaging
DL: Deep learning
EDV: End-diastolic volume
EF: Ejection fraction
ESV: End-systolic volume
FB: Free breathing
ICC: Intra-class-correlation coefficient
LV: Left ventricle
MRI: Magnetic resonance imaging
RV: Right ventricle
SAX: Short-axis stack
SV: Stroke volume
