# Supplementary material for: Pushing the limits of cardiac MRI: deep-learning based real-time cine imaging in free breathing vs breath hold
Source: Eur Radiol. 2025 Aug 23;36(2):1170–83. doi: 10.1007/s00330-025-11941-2 (PMC12953295; doi:10.1007/s00330-025-11941-2)

# Pushing the limits of cardiac MRI: deep-learning based real-time cine imaging in free breathing vs. breath hold

## ELECTRONIC SUPPLEMENTARY MATERIAL

*Table S1 MRI scanning parameter*

| MRI parameter | 3RR SonicDL (Reference) | 1RR SonicDL (breath hold/free breathing) |
|---|---|---|
| *Field of view [mm]* | 34x34 | 34x34 |
| *In-plane resolution [mm²]* | 1.7x1.5 | 1.9x1.9 |
| *Image pixel matrix* | 200x224 | 180x160 |
| *Slice thickness [mm]* | 8 | 8 |
| *Frames / cardiac cycle* | 30 | 30 |
| *TR [ms]* | 3.3 | 3.2 |
| *TE [ms]* | 1.2 | 1.2 |
| *Acceleration factor* | 6 | 12 |

| Bland-Altman Analysis | Reference vs. 1RR BH | | | Reference vs. 1RR FB | | | 1RR BH vs. 1RR FB | | |
|---|---|---|---|---|---|---|---|---|---|
| **Parameter** | *Bias* | *SD of bias* | *95% Limits of Agreement* | *Bias* | *SD of bias* | *95% Limits of Agreement* | *Bias* | *SD of bias* | *95% Limits of Agreement* |
| *LV EDV (ml)* | 3.1 | 5.9 | -8.6 – 14.75 | 2.2 | 9.1 | -15.6 – 19.9 | -0.9 | 9.9 | -20.3 - 18.5 |
| *LV ESV (ml)* | 1.2 | 4.3 | -7.1 – 9.6 | -1.1 | 7.2 | -15.3 – 13.1 | -2.3 | 7.5 | -17.1 – 12.4 |
| *LV SV (ml)* | 1.9 | 4.5 | -6.9 – 10.6 | 3.3 | 7.7 | -11.9 – 18.4 | 1.4 | 6.8 | -11.9 – 14.8 |
| *LV EF (%)* | -0.04 | 1.9 | -3.8 – 3.7 | -1.2 | 3.6 | -8.3 – 5.9 | 1.2 | 3.4 | -5.4 – 7.9 |
| *RV EDV (ml)* | 0.3 | 9.9 | -19.2 – 19.7 | -2.1 | 10.6 | -22.8 – 18.6 | -2.3 | 11.6 | -25.0 – 20.3 |
| *RV ESV (ml)* | -1.6 | 7.7 | -16.6 – 13.4 | -4.7 | 8.1 | -20.7 – 11.3 | -3.1 | 9.4 | -21.5 – 15.2 |
| *RV SV (ml)* | 1.8 | 6.6 | -11.0 – 14.7 | 2.6 | 8.4 | -13.8 – 19.1 | 0.8 | 7.5 | -13.9 – 15.5 |
| *RV EF (%)* | 1.1 | 3.0 | -4.7 – 6 | 2.2 | 3.6 | -4.8 – 9.1 | 1.1 | 3.2 | -5.1 – 7.3 |

**Supplemental Table**

Table S2: Bland-Altman Analysis of all left ventricular (LV) and right ventricular (RV) volumetric parameter. Analysis of 1RR_BH vs. Reference, 1RR_FB vs Reference and 1RR_BH vs 1RR_FB of left ventricular volumetric parameter. EDV = end- diastolic volume, ESV = end-systolic volume, SV = stroke volume, EF = ejection fraction. FB= Free Breathing, BH = Breath Hold

**Figure S1:** Examples of different subjective images quality ratings.

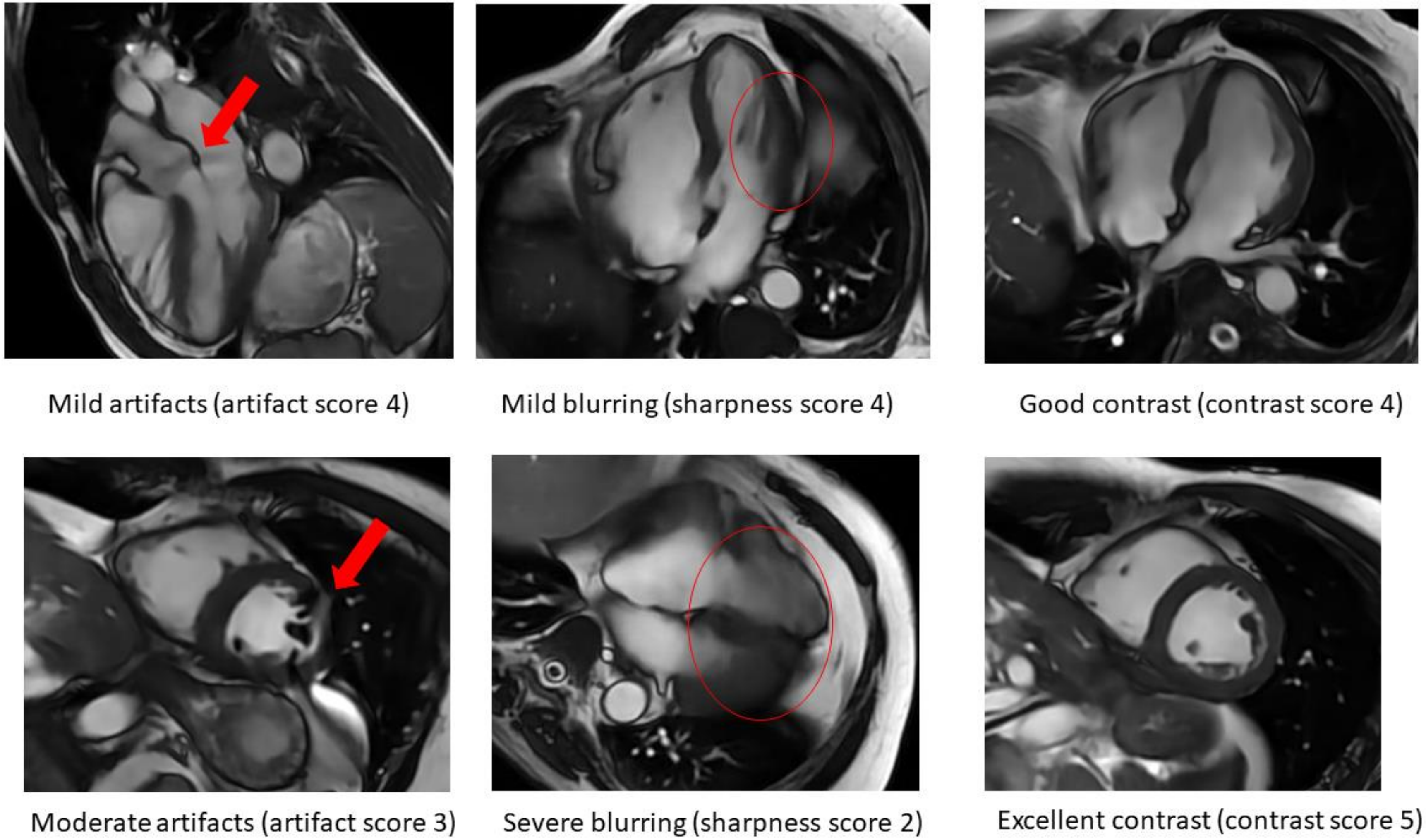

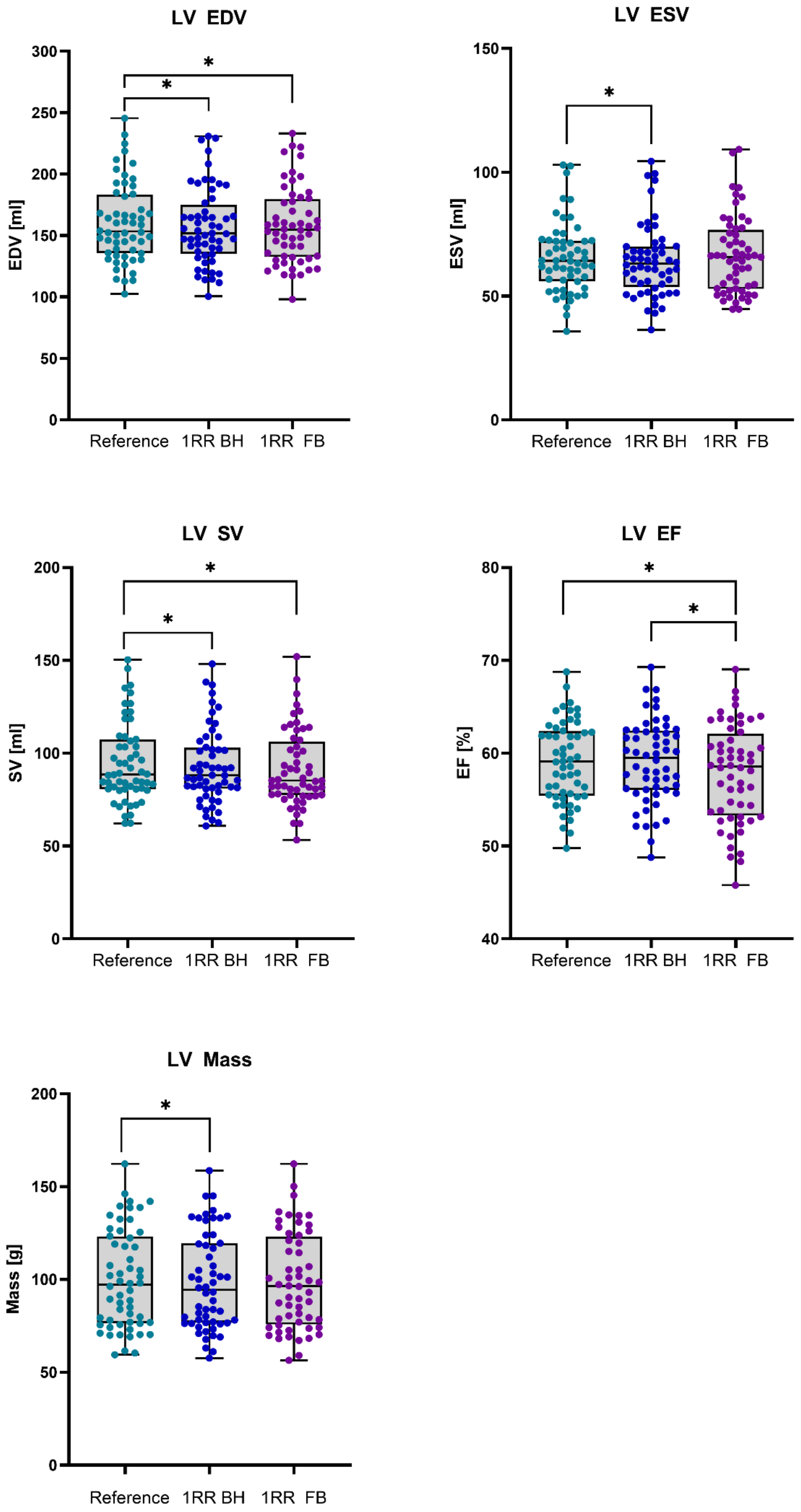


**Figure S2**: Boxplots of volumetric parameter of the left ventricle acquired for the reference, the 1rr breath hold (BH) and the 1RR free breathing (FB) sequence with * p<0.001 from Wilcoxon paired signed rank sum test. EDV = end-diastolic volume, ESV = end-systolic volume, SV = stroke volume, EF = ejection fraction

**Figure S3**: Boxplots of volumetric parameter of the right ventricle acquired for the reference, the 1rr breath hold (BH) and the 1RR free breathing (FB) sequence with * p<0.001 from Wilcoxon paired signed rank sum test. EDV = end- diastolic volume, ESV = end-systolic volume, SV = stroke volume, EF = ejection fraction

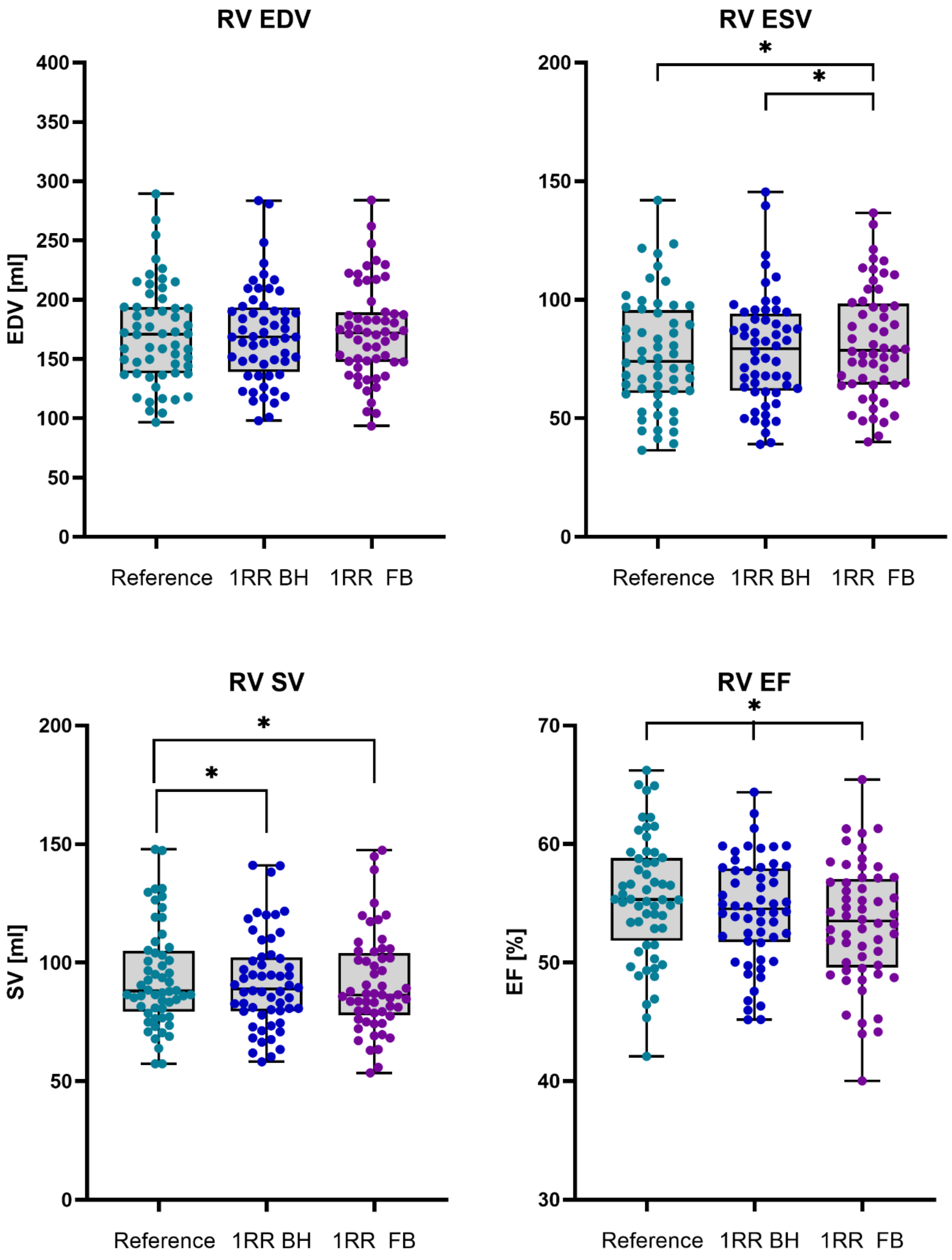

**Figure S4**: Bland-Altman Plots of all LV volumetric parameter. Analysis of 1RR_BH vs. Reference, 1RR_FB vs Reference and 1RR_BH vs 1RR_FB of left ventricular volumetric parameter. EDV = end- diastolic volume, ESV = end-systolic volume, SV = stroke volume, EF = ejection fraction. FB= Free Breathing, BH = Breath Hold

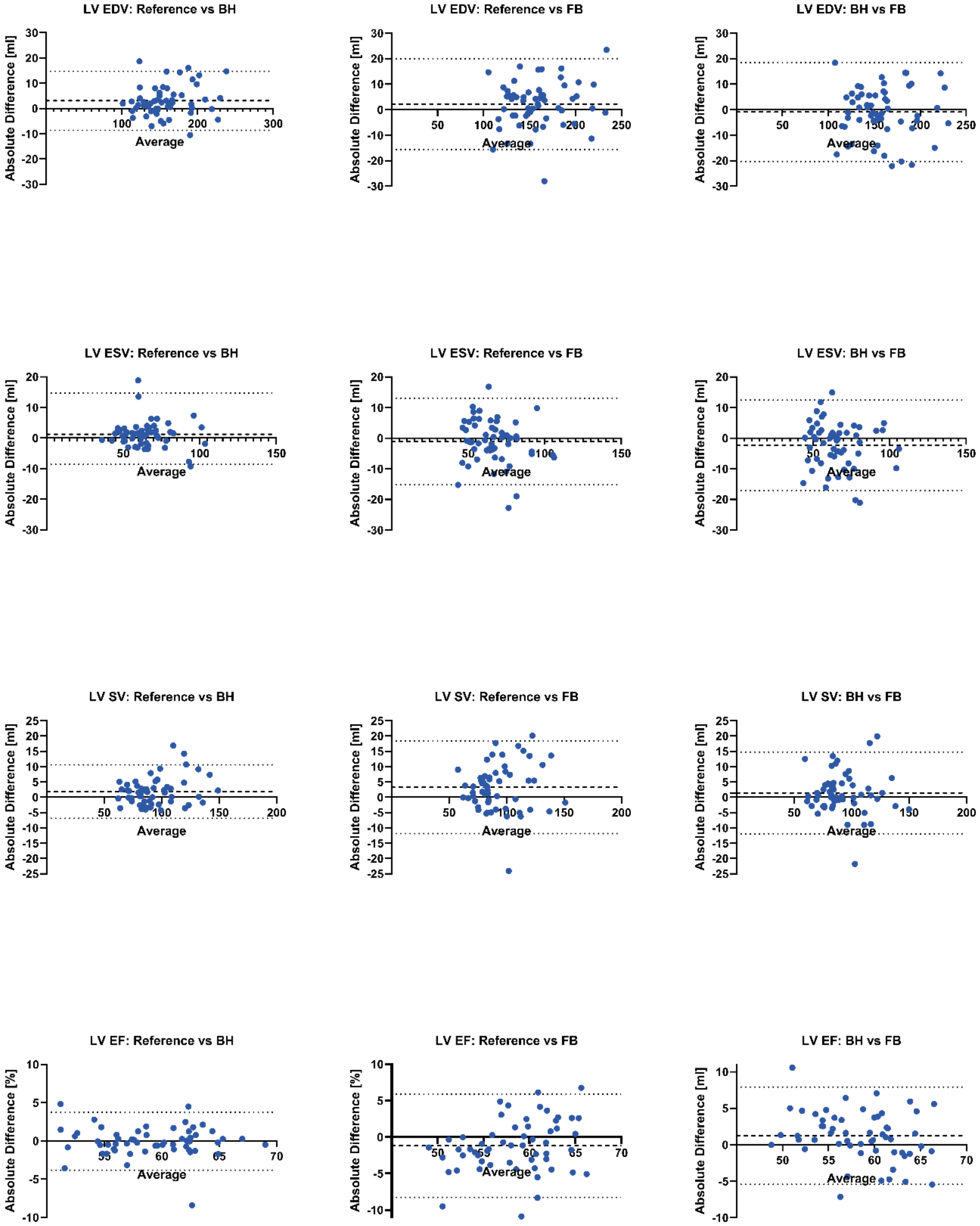

Supplement: Supplementary file 1 — ELECTRONIC SUPPLEMENTARY MATERIAL [file 330_2025_11941_MOESM1_ESM.pdf]
